# Clinical features associated with the development of hydrocephalus following TBI in the paediatric age group

**DOI:** 10.1007/s00381-020-04764-7

**Published:** 2020-06-29

**Authors:** Ronak Ved, Rebecca Fraser, Sarah Hamadneh, Malik Zaben, Paul Leach

**Affiliations:** Department of Neurosurgery, University Hospital Wales, 4th Floor, Heath Park Way, Cardiff, CF14 4XW UK

**Keywords:** Hydrocephalus, Paediatric, Trauma, Injury

## Abstract

**Introduction:**

Predictive factors for post-traumatic hydrocephalus (PTH) in adults have been elucidated but remain uncertain for children. We aimed to identify the prevalence of PTH in paediatric patients and identify clinical/radiological factors which may increase the probability of children developing PTH.

**Methods:**

This was a retrospective study of all patients < 16 years old admitted to our unit with traumatic brain injury (TBI) between March 2013 and June 2018, 108 patients in total. Patients were classified as mild (13–15), moderate (9–12) or severe (3–8) TBI based on admission GCS. Three independent reviewers collected data from case notes. CT scans were reviewed for hydrocephalus using Evan’s index. Two-tailed Fisher’s exact tests with a *p* value < 0.05 were considered statistically significant.

**Results:**

Median patient age was 7 years, and 65% were males (*n* = 70). PTH wasn’t identified in any patients with mild/moderate TBI (*n* = 79). In cases of severe TBI (*n* = 29), three patients developed PTH requiring ventriculoperitoneal shunting (10%; *p* = 0.02). Radiological features which were significantly more common in the PTH group were intraventricular haemorrhage (*p* = 0.05) and subarachnoid haemorrhage (*p* = 0.03). There was also a trend towards a statistically significant association with subdural haematoma (*p* = 0.07). The need for other neurosurgical procedures, such as fracture elevation and craniotomy, did not increase the probability of developing with PTH (*p* = 0.08).

**Discussion:**

The prevalence of PTH in our study is 2.7%. Factors which may be associated with a higher probability of developing PTH may include IVH, SAH, severity of TBI, and subdural haematoma. We propose a national prospective multicentre database of paediatric PTH. The data collected on prevalence, presentation, risk factors, and management could guide contemporary management and improve the outcomes of children with PTH.

## Introduction

Traumatic brain injury (TBI) is a leading cause of death and disability in young people in the world [1, 2]. Post-traumatic hydrocephalus (PTH) affects 10–40% of adult patients after severe TBI [3]. It is associated with poor clinical outcomes but is also amenable to surgical treatment, such as CSF shunting [4]. Factors such as injury severity, the presence of subdural haematomas, and the need for surgery have been associated with an increased risk of adult patients developing PTH [5–8].

Many studies around PTH have assessed adult patients; however, their findings may not necessarily correlate with PTH in the paediatric patient group. There is thus a clinical need to identify the prevalence of PTH in paediatric patients and to assess which factors may predispose patients to the debilitating, but potentially treatable, development of PTH.

## Objectives

The objectives of the present study were to:

1. Ascertain the prevalence of hydrocephalus in paediatric traumatic head injury patients at a single neurosurgical unit
2. Identify potential clinical or radiological features which may predict the development of post-traumatic hydrocephalus in children

## Methods

**Fig. 1:**
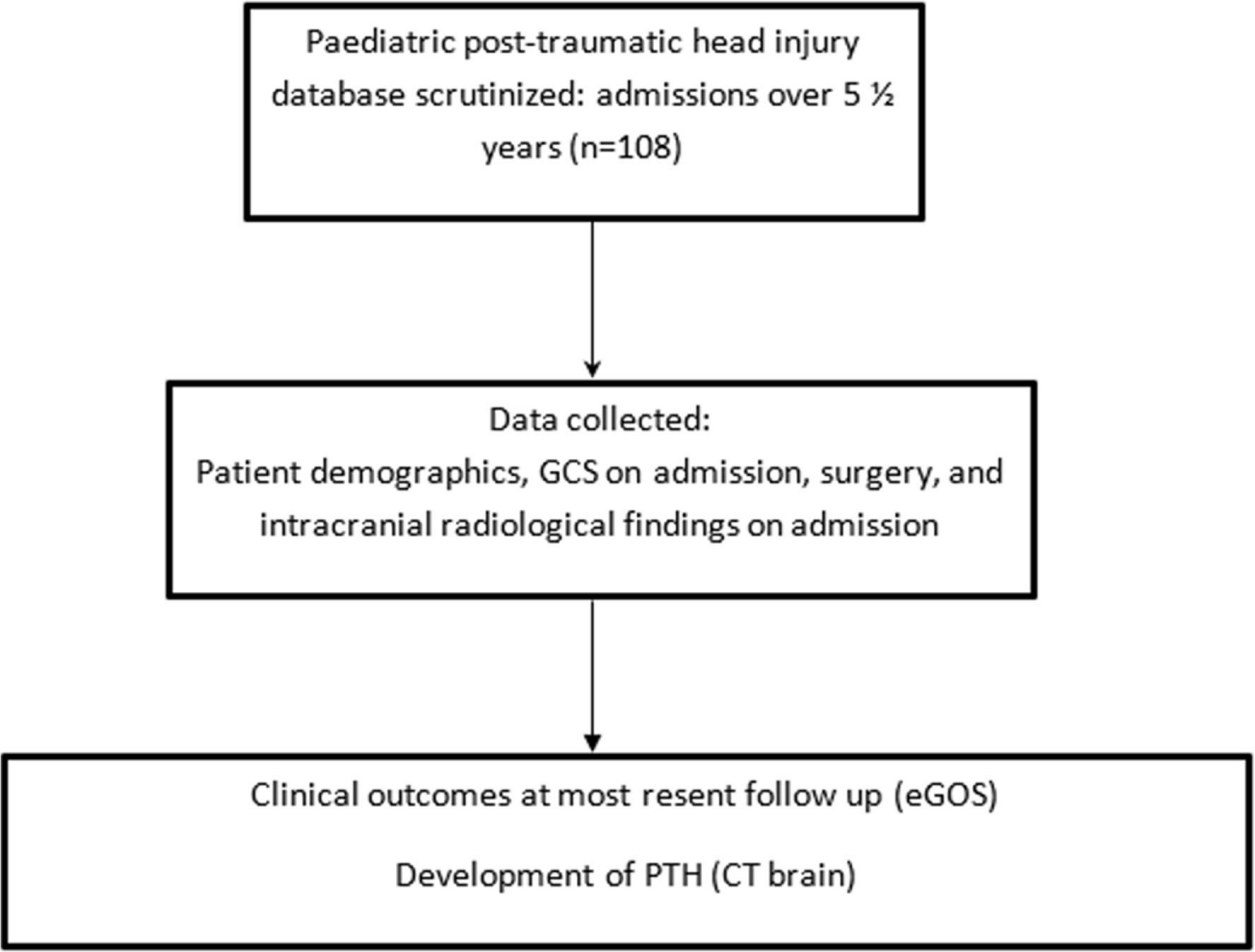
Method flow chart outlining the participant selection process for the present study. Clinical and radiological findings assessed by the investigators were selected based upon factors listed within the current literature that may predispose adult patients to PTH [5], alongside any other notable features or pathological findings for each patient. The comprehensive list of features assessed for is provided in the results in Table 2

Participants were selected via scrutiny of a prospectively-maintained paediatric TBI database at a single neurosurgical unit. This enabled the study to capture all patients under the age of 16 admitted to a national neurosurgical unit with TBI between March 2013 and June 2018. Electronic and paper-based case notes were then reviewed by three independent investigators. Figure 1 outlines the patient selection process and the data collection processes that were undertaken.

Demographic, clinical, and imaging data at admission, during the hospital stay, and at follow-up were used for this study. All CT scans were reviewed for PTH using the Evan’s index and criteria described by Gudeman et al. [2]. For statistical analysis of categorical data, two-tailed Fisher’s exact tests with a *p* value < 0.05 were considered statistically significant.

## Results

**Fig. 2:**
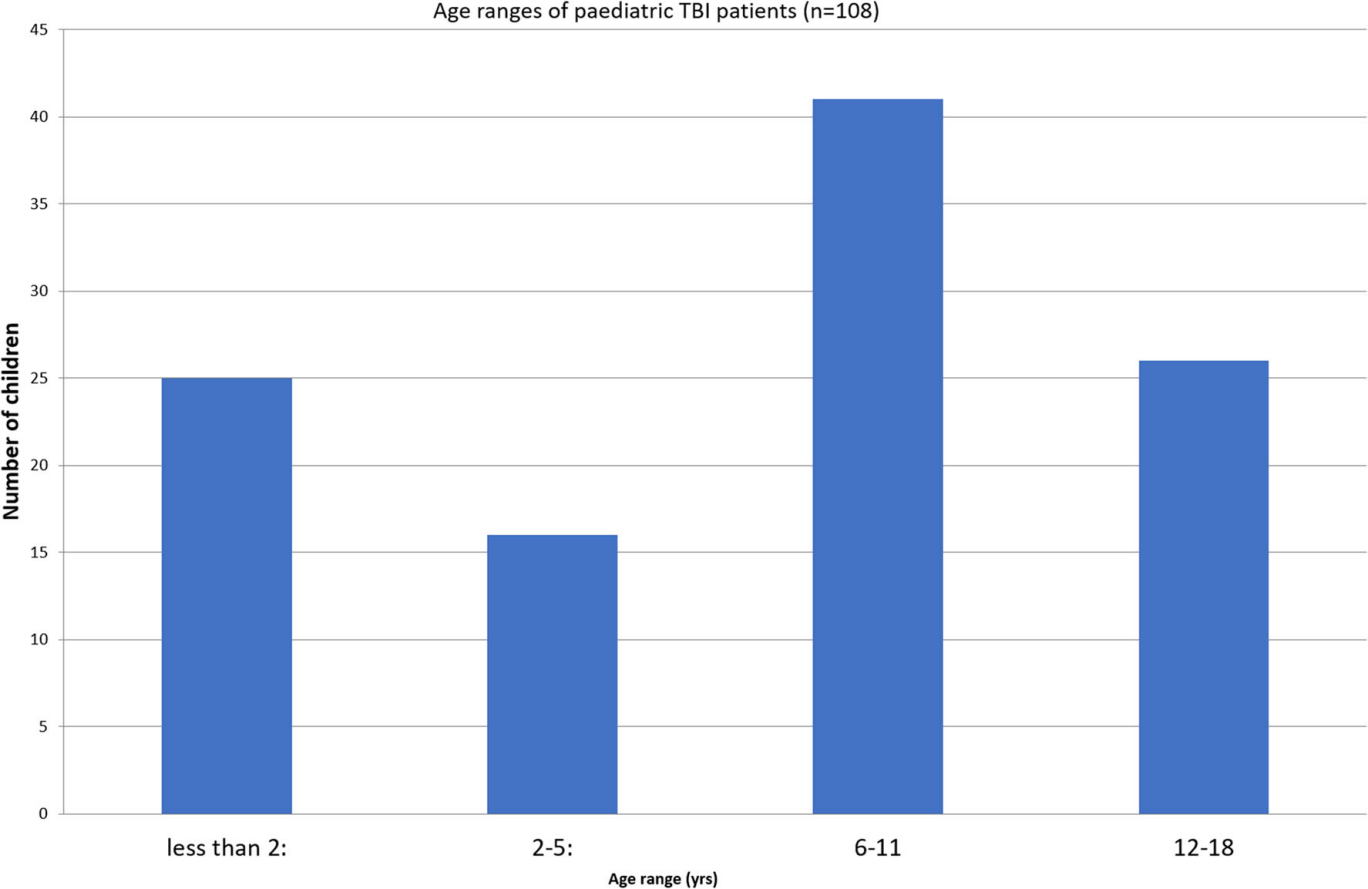
Age range distribution for TBI across the paediatric cohort. The most common cohort ages for TBI were 6–11 years and 12–18 years

One hundred eight patients were included in the study analysis. The mean age of the cohort was 7 years and 5 months (11 days–15 years, 9 months). Thirty-five percent of the cohort were female (*n* = 38), and 65% (*n* = 70) were male. Patient ages were categorized into four age groups: < 2 years, 2–5 years, 6–11 years, and 12–16 years. The most common age bracket for TBI was 6–11 years (Fig. 2). The most common mechanisms of injury were falls and road traffic collisions (Fig. 2). No patients in the study cohort died as a result of their TBI. Median follow-up for the study cohort was 48 months.

**Fig. 3:**
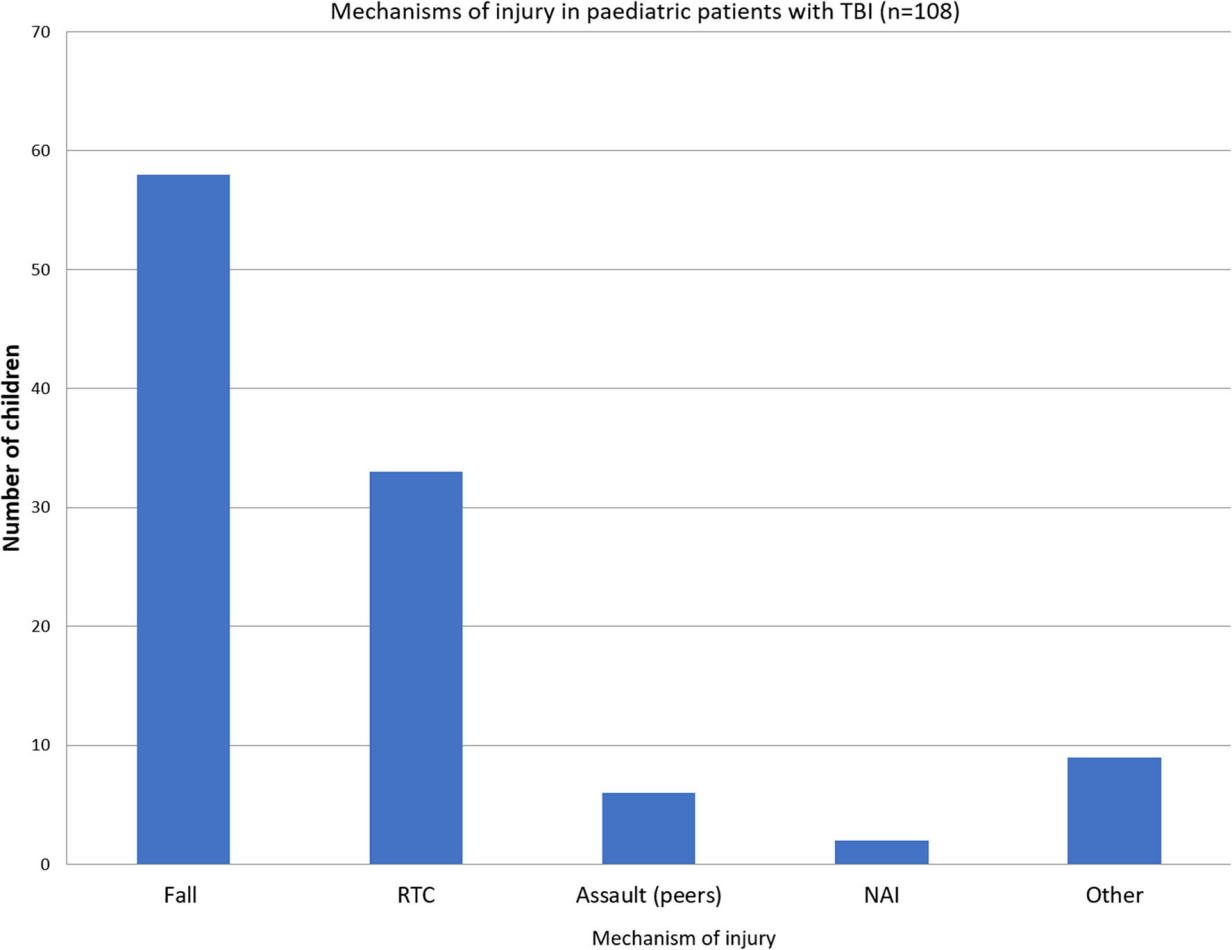
Mechanism of TBI for paediatric patients. The most common MOIs were falls (51%; *n* = 55) and RTC (30%; *n* = 32). ‘Other’ injuries included objects falling onto children and injuries from animals. *RTC* road traffic collision, *MOI* mechanism of injury, *NAI* non-accidental injury

**Fig. 4:**
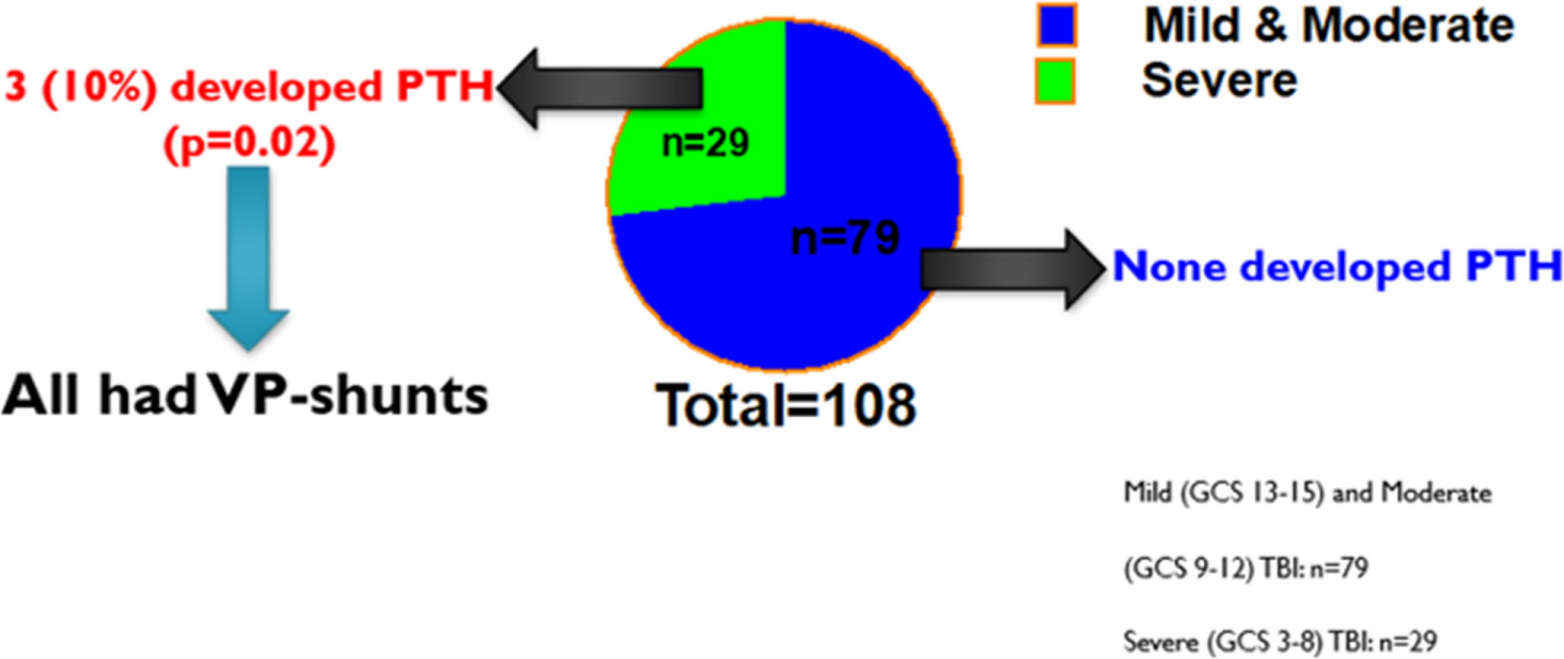
The proportion of paediatric TBI patients presenting with mild and moderate TBI vs those presenting with severe TBI. All patients who developed PTH had suffered a severe head injury, and all three required insertion of a ventriculoperitoneal shunt. *GCS* Glasgow Coma Scale, *PTH* post-traumatic hydrocephalus

TBI patients where next dichotomized (Fig. 3) into a mild and moderate TBI cohort (admission GCS > 8; *n* = 79) and a severe TBI cohort (admission GCS 8 or lower; *n* = 29). No patients in the mild/moderate TBI group developed PTH, whereas three patients who suffered a severe TBI developed PTH (10%; *p* = 0.02) (Fig. 4).

Since PTH was only seen amongst patients who had suffered serve TBI, this subcohort underwent analysis to identify features which may increase the probability of developing PTH (Table 1). None of the severe TBI patients had evidence of ventriculomegaly on their admission scan. Seventeen of these patients underwent emergency craniotomy, and a further seventeen required other forms of emergency neurosurgery (e.g. elevation of depressed skull fractures, wound washouts, burr hole evacuations of haematomas). One of the patients who underwent emergency neurosurgery (craniotomy for evacuation of a subdural haematoma) went on to develop PTH (Tables 1 and 2). The need for emergency neurosurgery of any kind did not predict the development of PTH (*p* = 0.43).

**Table 1:** List of clinical radiological features present on admission to hospital and on initial CT head scans of paediatric patients following severe TBI (GCS 8 or lower on admission)

Clinical/radiological feature	No PTH (*n* = 26)	PTH (n = 3)
Emergency craniotomy/craniectomy	16 (61%)	1 (33%)
Other emergency operation	17 (65%)	0 (0%)
Ventriculomegaly on initial scan	0 (0%)	0 (0%)
IVH	3 (12%)	3 (100%)
SAH	7 (27%)	3 (100%)
IVH + SAH	2 (8%)	3 (100%)
Skull fracture	13 (50%)	3 (100%)
SDH	10 (39%)	3 (100%)
EDH	8 (31%)	1 (33%)
Cortical contusions	4 (15%)	0 (0%)
Midline shift	3 (12%)	1 (33%)
Effacement of basal cisterns	3 (12%)	0 (0%)
Petechial haemorrhage	3(12%)	0(0%)

*IVH* intraventricular haemorrhage, *SAH* subarachnoid haemorrhage, *SDH* subdural haematoma, *EDH* extradural haematoma, *GCS* Glasgow Coma Scale

The radiological features at the time of their injury for all three patients who developed PTH are outlined in more detail in Table 2. IVH and SAH were significantly associated with subsequent PTH, whilst there was also a trend towards significance for association between SDH with the development of PTH (Table 2).

**Table 2:** Radiological features associated with PTH in the study cohort

Radiological feature	No of patients	PTH	No PTH	*p* value
IVH	6	3	3	0.05
SAH	10	3	7	0.03
SAH + IVH	5	3	2	< 0.01
SDH	13	3	10	0.07
Skull fracture	16	3	13	0.2
Effacement of BS	2	0	2	1
Midline shift	4	1	3	1

All three patients with PTH suffered a skull fracture, IVH, SAH, and SDH. The association between PTH and initial IVH and SAH (particularly when both were present together) reached statistical significance. Fisher’s exact test was used for statistical analysis, with *p* values < 0.05 being considered significant

*BS* basal cisterns

Two of the patients who developed PTH were 9 years old at the time of their injury. One was aged 10 months. Two were female (age 9 years and 10 months), and one was male (age 9 years). The mechanisms of injury were one road traffic collision, one fall from height, and one hit by a large falling object. Whilst all three suffered a skull fracture, none of these were depressed fractures. Only one patient underwent emergency surgery (Table 3). PTH developed between 1 and 3 months post-injury, with presenting symptoms and signs including persistent headaches, slow neurological recovery, and drowsiness, respectively, amongst the three patients. All three patients underwent insertion of VP shunt upon diagnosis of PTH, all achieving satisfactory CSF diversion based on postoperative head scans and improvements in their clinical symptoms. None of the cohort has required revision of their VP shunt up to the date of submission (follow-up periods of 3, 4, and 5 years, respectively; Table 3). Extended Glasgow Outcome Scores ranged from 8 (upper good recovery) to 5 (moderate disability).

**Table 3:** Clinical features, radiological details, and clinical outcomes for all patients who developed PTH in the study cohort

Age	Skull fracture	SDH size	SDH location	Interval (injury to PTH)	Other surgery	CSF diversion	eGOS
9 years	Left frontal bone	4 mm	Left temporal + interhemispheric	1 month	Nil	VP shunt (0 revisions)	8
	Left occipital bone						
	Right parietal bone (non-depressed)						
9 years	Left temporal bone	12 mm	Left convexity	1 month	Craniotomy	VP shunt (0 revisions)	5
	Left occipital bone (non-depressed)						
10 months	Left occipital bone	4 mm	Right temporal + adjacent to torcula	3 months	Nil	VP shunt (0 revisions)	6
	Right temporal bone						
	Right occipital bone (non-depressed)						

None of this subcohort suffered a depressed skull fracture. The interval between injury and the development of PTH was between 1 and 3 months post-TBI. One patient underwent emergency surgery prior to their VP shunt insertion. No patients have required revision of their VP shunts, and eGOS ranged between 5 and 8

*eGOS* Extended Glasgow Outcome Scores, *VP shunt* ventriculoperitoneal shunt

## Discussion

PTH is a significant cause of disability in adults after TBI [9]. It may prolong hospital stay, worsen morbidity, and raise hospital costs [10]. Although the characteristics of PTH in adults have been delineated, there is a relative lack of knowledge regarding PTH in children [2, 11]. The ability to identify children who may be most at risk of developing PTH would be valuable to clinicians for guiding management and prognostication following paediatric TBI. To this end, the present study aimed to identify the prevalence of PTH in a paediatric neurosurgical population and begin to investigate factors which may help to increase the suspicion of clinicians for subsequent development of PTH.

The prevalence of PTH in this study was just under 3%, rising to 10% when severe TBI is analysed as a subcohort. These figures are lower than those reported in the literature for adults, which range from 10 to 50% [1, 5, 6, 8, 12]. However, the identified prevalence is higher than the reported frequency of PTH in two recent retrospective studies of paediatric PTH, which report prevalence rates of ~ 1% [5, 10]. These large studies are large database reviews of paediatric head injury admissions across multiple healthcare sites. These reviews thus canvassed large numbers of patients, (~ 100,000 each) and reviewed electronic records to identify clinical features which may have associations with PTH. Both studies identified similar prevalence rates of PTH in children and similar clinical features associated with PTH, such as younger age [5] and the need for other neurosurgical procedures [10]. Rumalla et al. also reviewed clinical pathologies that were shown to be associated with PTH, which included SAH and SDH [10]. The present study also identified that these features were either significantly associated with PTH (SAH) or demonstrated a trend towards an association (SDH). The focus of the present study was to review radiological features which may raise the probability of PTH in children, which was not the focus of the two larger studies [5, 10].

One explanation for the prevalence rates identified in the present study is that, to our knowledge, this is one of the first studies in which there has been a focused assessment of the prevalence of PTH in children admitted to a single paediatric neurosurgical centre only. The two previous, albeit larger, studies pooled patients from a wide range of hospitals and primary care settings [5, 10]. This may have diluted the prevalence of the condition, with large numbers of mild and moderate TBI patients masking a higher prevalence of PTH amongst children with more severe brain injuries who are admitted directly to neurosurgery units. Our study and others have purported that severe TBI patients are the group most likely to develop PTH [4]. Furthermore, the two larger studies of PTH in children thus far defined paediatric populations as < 21 years old, which is an older cut-off compared with that used in the UK (< 16 years old). The average age of patients in these large cross-sectional studies was thus older than in the present work (12 years vs 7 years). Finally, the cross-sectional population-based studies provide snapshot, retrospective data, with short follow-up periods of around 12 months [5, 8, 10]. The authors of one of the studies remind readers that their databases are only reliable for reviewing inpatient stays, complications, and outcomes, meaning that patients who developed PTH after being discharged from their initial hospital admission will not have been captured using their methodology [10]. Our study group was a smaller, but focused cohort of children admitted to a neurosurgical unit. Our longer median follow-up data (median 48 months) helps support the notion that PTH is amenable to treatment by VP shunting and corroborates other studies’ inferences that long-term outcomes are improved for PTH patients by CSF diversion [7, 13].

In adult patients, the factors that have been associated with the development of PTH include younger age [7, 13, 14], the presence of subdural haematoma [8, 10], and the need for decompressive craniectomy [7]. However, these factors have not been consistently demonstrated in all case series, with some even reporting opposite associations, such as one study which identified older age as a risk factor for PTH [9]. Nonetheless, the factors identified in this study that may increase clinical suspicion of subsequent development of PTH are severity of TBI, IVH, SAH, and the combination of IVH and SAH together. There was also a trend towards an association between subdural haematoma and a higher probability of PTH. All these proposed risk factors have been identified in other works in the adult and paediatric PTH literature [3, 5, 7, 8, 10, 15, 16]. These seem reasonable factors to predict PTH, given that injury severity and the volume of intracranial blood load is associated with the development of hydrocephalus in other pathologies, such as non-traumatic subarachnoid haemorrhage [3].

Our study did not identify emergency neurosurgery, such as craniectomy, as increasing the probability of PTH. However, decompressive craniectomy (DC) has been associated with up to a 50% risk of development of PTH in adults [6–8]. The risk of PTH is even higher in adult DC patients who also have or develop subdural haematomas/hygromas [8]. The small sample size of the present study may explain why we did not identify DC as a risk factor in our study population; only three patients required a DC. As a single paediatric neurosurgery centre, we are unlikely to improve the power of future studies in a timely manner with our current patient workload. Therefore, the most efficient way to maximize the power of much-needed follow-up studies, without diluting study populations with non-neurosurgical unit data and avoiding the snapshot nature of cross-sectional methodologies, will be to design a multicentre, prospectively maintained national database of paediatric TBI. This database could cover admissions, management, and complications of children who have suffered a head injury severe enough to warrant referral or admission to a neurosurgical unit throughout the country. Such a database could drastically improve the ability of clinicians to mine valuable data to investigate prognostic factors and guide management of paediatric TBI. This would include, but not necessarily be limited to, formally defining the predictive factors and optimal treatment strategies for paediatric PTH.

## Conclusion

The prevalence of PTH in this study’s neurosurgical paediatric population was 2.7%, which is higher than that in previous studies of paediatric TBI. Potential risk factors which may increase the probability of the development of PTH in children may include low GCS (i.e. severity of TBI) and the presence of intracranial blood load in the form of IVH, SAH, and possibly SDH. A larger cohort of paediatric TBI patients admitted to neurosurgery should be analysed to elucidate predictive factors for PTH. We propose a national, prospective, multicentre evaluation of paediatric PTH prevalence, presentation, risk factors, and management. This evidence could inform the contemporary management and improve the clinical outcomes of children with PTH.

## Data Availability

The datasets generated during and/or analysed during the current study are available from the corresponding author on reasonable request.
